# Melodic Universals Emerge or Are Sustained Through Cultural Evolution

**DOI:** 10.3389/fpsyg.2021.668300

**Published:** 2021-08-02

**Authors:** Tessa Verhoef, Andrea Ravignani

**Affiliations:** ^1^Creative Intelligence Lab, Leiden Institute for Advanced Computer Science, Leiden University, Leiden, Netherlands; ^2^Comparative Bioacoustics Group, Max Planck Institute for Psycholinguistics, Nijmegen, Netherlands

**Keywords:** cultural transmission, music, melodic universals, iterated learning, continuous signals

## Abstract

To understand why music is structured the way it is, we need an explanation that accounts for both the universality and variability found in musical traditions. Here we test whether statistical universals that have been identified for melodic structures in music can emerge as a result of cultural adaptation to human biases through iterated learning. We use data from an experiment in which artificial whistled systems, where sounds produced with a slide whistle were learned by human participants and transmitted multiple times from person to person. These sets of whistled signals needed to be memorised and recalled and the reproductions of one participant were used as the input set for the next. We tested for the emergence of seven different melodic features, such as discrete pitches, motivic patterns, or phrase repetition, and found some evidence for the presence of most of these statistical universals. We interpret this as promising evidence that, similarly to rhythmic universals, iterated learning experiments can also unearth melodic statistical universals. More, ideally cross-cultural, experiments are nonetheless needed. Simulating the cultural transmission of artificial proto-musical systems can help unravel the origins of universal tendencies in musical structures.

## Introduction

Why is music the way it is? As for many human traits, music has also been the object of a nature-nurture debate. Some disciplines studying music, such as psychology, neuroscience, and biology, often highlight which musical features are present in most cultures and what neurobiological traits for musicality are shared among all humans. Other disciplines, including anthropology and the humanities, prefer instead to highlight the cultural variability and uniqueness of each musical event. While the former scholars focus on universality, the latter focus on diversity. The two approaches can be reconciled in several ways (Savage, 2019; Shanahan and Albrecht, 2019; Jacoby et al., 2020). One of these approaches, adopted here, is to show how nature and nurture sustain each other to give rise to human music: Statistical universals can emerge via human cognitive biases amplified and modulated by culture.

Historically, the concept of “universals” has been much explored and debated especially in linguistics. In this field, a “linguistic universal” can have several meanings. In one of its simplest forms, an implicational universal could be: if a language has a specific sound X, it will also have a sound Y (Greenberg, 1963). Alternatively, an absolute universal is a feature found in all natural languages used by humans. For instance, one could say that all languages are spoken: a false example of a universal, because many languages are signed. In fact, some scholars have doubted the usefulness and existence of absolute universals in languages altogether (Evans and Levinson, 2009). The last decades have seen increased focus on language diversity, rather than universality (Evans and Levinson, 2009), making statistical universals (i.e., universal tendencies) the main tenable and empirically testable approach. A statistical universal, in fact, is a feature which appears very frequently and above chance across languages.

The concept of universals, needless to say, can be applied not only to languages, but also to other human cultural artefacts. A prime example of purported universal features may be found in music. Surprisingly, only a few years ago were musical universals thoroughly coded and their presence across cultures tested empirically. Savage et al. (2015) showed that there are 18 musical features which can be found, above statistical chance level, across musical cultures. Some of these features concern musical context while most are applicable to acoustic features, such as rhythm, timbre, melody and harmony (Savage et al., 2015).

In parallel to this work, psychological research has tackled the mechanisms involved in the emergence of individual musical features. These experiments, inspired by much language research (Kirby et al., 2008; Verhoef, 2012; deCastro-Arrazola and Kirby, 2019), have later been adapted to music (Ravignani et al., 2016; Lumaca and Baggio, 2017). The fundamental idea here is to replicate with experimental participants what culture may otherwise accomplish over much longer timescales: the experimenter asks a participant to learn and copy a behaviour which was produced by another participant who learnt it in the same way. Over time, small biases get amplified by this transmission process, leading to measurable patterns which can be attributed to a combination of human cognitive propensities and cultural transmission.

Experimental cultural transmission of musical features is still understudied but can already count on a few empirical results. Ma et al. (2019), for instance, used this method to show how vocalisations copied from one person to another, and so on, diverge into two strains, music-like and speech-like, depending on the referential context they are used in. Others have tackled rhythmic universals, showing that transmission of randomly timed percussive events leads to sequences becoming more rhythmic; this convergence towards rhythmicity occurs even when participants are unknowingly copying their own sequences and it generalises across cultures (Ravignani et al., 2016, 2018; Jacoby and McDermott, 2017). Notably, cultural transmission of drumming patterns can indeed result in rhythmic sequences containing all six universals for musical rhythm (Savage et al., 2015; Ravignani et al., 2016). Notice that these universals need not be cognitively driven or a result of neural specialisation, but can also arise via motoric constraints and affordances (Miton et al., 2020).

Along all previous research, Lumaca and Baggio (2016, 2017, 2018) and Lumaca et al. (2021) performed a series of studies which pioneered iterated learning of melodic sequences. In their studies, Lumaca and Baggio (2016, 2017, 2018) showed how some melodic universals emerge or are processed in the brain. However, some universals could not be tested in Lumaca and Baggio (2017) because the sound signals presented to participants were already discretized and constrained since they consisted of a fixed set of only five tones produced with keys. Inspired by this work, we hypothesised (Ravignani and Verhoef, 2018) that potentially all melody-related universals could emerge in a cultural transmission task if participants were given continuous signals to start with. The data we present here aims at testing this hypothesis empirically.

According to the statistical universals found in the pitch domain (Savage et al., 2015), and the parallel evidence for cultural transmission converging to rhythmic universals, we hypothesise that cultural transmission can also lead to seven melodic universals (Melodic Universals, MU1-7 below). In particular, we predict that continuous, culturally-transmitted signals would become discretized (MU1), that is, transition from continuous, modulated frequencies to discrete pitches. These discrete pitches would be organised in scales of few (≤ 7) elements per octave (MU2). Melodies constructed from these pitches should show descending or arched contours (MU3), and these melody contours would span small frequency intervals (MU4). Overall, the resulting proto-musical system would include motivic patterns (MU5), featuring repetition (MU6), and short phrases (MU7) (Savage et al., 2015; Ravignani and Verhoef, 2018).

The design of Lumaca and Baggio (2016, 2017) was quite innovative and allowed to test several hypotheses. However, the sequences participants were exposed to already contained discrete pitches, preventing the authors from testing some of the universals. In fact, Lumaca and Baggio's data (Lumaca and Baggio, 2017) provide some evidence for (MU3), (MU4), and (MU6), leaving the other features unexplored (Ravignani and Verhoef, 2018).

Here we measure the emergence of melodic universals with a dataset that was collected in the context of language research. Our set contains continuous signals with varying pitch, which makes it ideal for analyses of statistical universals in melodic patterns. In brief, having targeted a set of melodic universals, we use a dataset collected for non-musical purposes to test whether even when participants are not prompted towards music, melodic universals emerge nonetheless.

## Materials and Methods

### Overview

**Figure 1:**
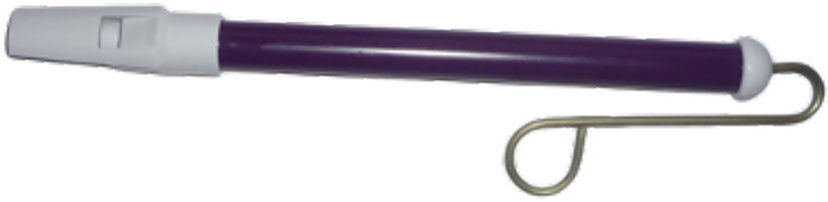
Slide whistle (reproduced with permission from Verhoef, 2012).

The data used for our analyses was collected as part of a study which was originally designed to investigate the emergence of combinatorial structure in language (Verhoef, 2012; Verhoef et al., 2014). In this experiment, participants learned and reproduced sets of whistle sounds using a slide whistle (plastic version by Grover-Trophy, see Figure 1). Given the fact that this study involved the production of continuous acoustic signals and the use of a musical instrument, these results are relevant for discussions on the emergence of melodic patterns in music as well. The slide whistle has a plunger that can be used to adjust the pitch of the whistle sounds within a range of between about 450 and 2500 Hz.

### Set-Up

During the experiment, participants were asked to memorise and reproduce a set of 12 different whistle sounds. They completed four rounds of learning and recall. In the learning phase they were exposed to all 12 signals one by one, and asked to imitate each sound with the slide whistle immediately. After this, a recall phase followed in which they reproduced all 12 whistles in their own preferred order from memory. The input stimuli of one participant consisted of the output that the previous participant produced in the last recall round (or the initial input set). Transmission was continued in this manner until there were 10 participants in each chain and four parallel chains were completed. The resulting data therefore consist of 40 sets of 12 whistles, each produced by 40 different participants, in different locations across the four transmission chains. Full details of the experimental procedure can be found in Verhoef et al. (2014).

### Participants

All participants were university students from either the University of California San Diego, or the University of Amsterdam, ranging in age from 18 to 32 (with a mean of 22). Twenty-six were female. Each chain contained either three or four male participants. They were paid 10 euros (Amsterdam) or 10 US dollars (San Diego) in cash for their time. The study was approved by the ethics committee of both institutions. Participants were asked whether they had any experience in music, such as playing an instrument or musical training and 65% of all participants answered “yes” to this question. Per chain, this resulted in 5 out of 10 in chain one, 9 out of 10 in chain two and 6 out of 10 in chains three and four. To make sure musical training did not have any influence on the transmission of the whistles, we used a linear mixed-effects model to predict reproduction error on the basis of generation ^*^ musical training. We found an effect of generation on reproduction error [as was found in the original paper by Verhoef et al. (2014)] but no effect of musical training and no interaction between generation and musical training.

### Initial Whistle Set

**Figure 2:**
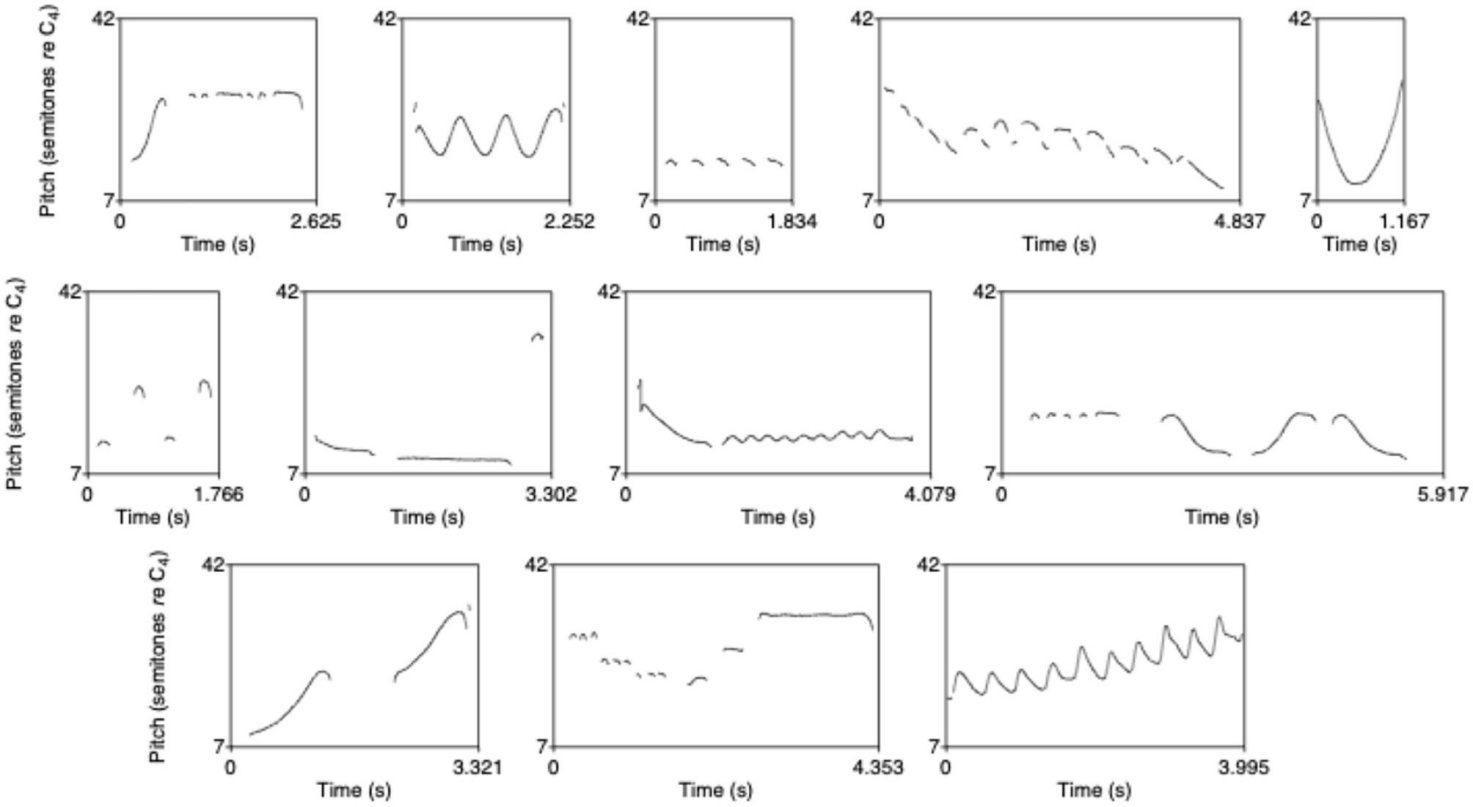
Whistles from the initial whistle set, plotted as pitch tracks on a semitone scale (From Verhoef, 2013).

As described in Verhoef et al. (2014), the initial set of whistles that was given to the first participant of each chain contained sounds that were created by multiple persons (two sounds from the same person at the most) who participated in an early exploratory pilot study and were asked to freely record at least 10 whistle sounds. The sounds exhibited many different “techniques” of whistling (such as staccato, glissando, siren-like, smooth or broken) with as little as possible reuse of basic elements. Figure 2 shows the complete set of 12 whistles plotted as pitch tracks on a semitone scale using Praat (Boersma, 2001).

### Whistle Data Pre-processing

**Figure 3:**
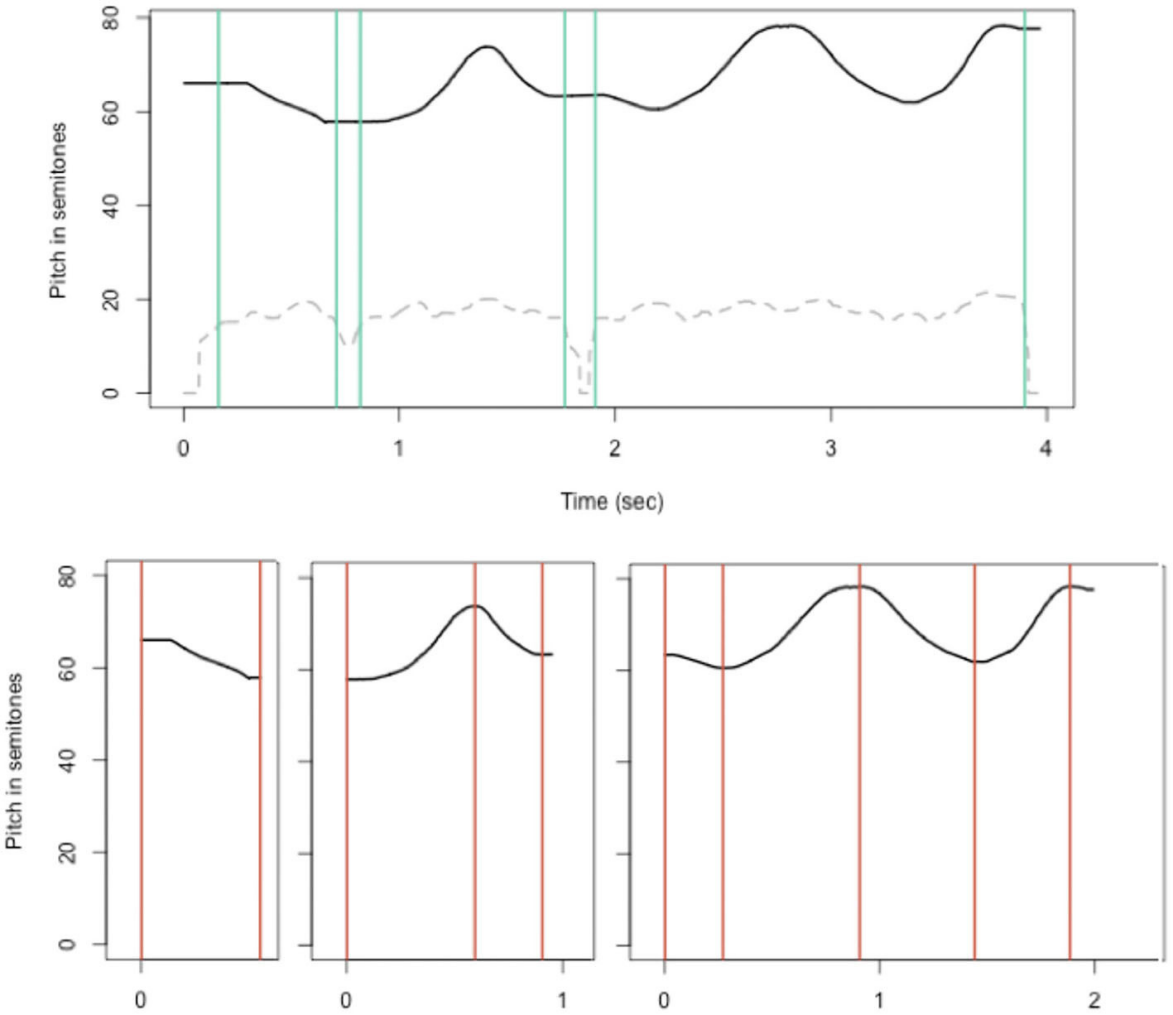
A whistle, plotted as pitch track on a semitone scale (black line) segmented into sounding and silent parts (borders shown in green vertical lines), based on the intensity track as shown by the dashed grey line [ranging from 0 (silent) to 92.6, actual value divided by 4 to fit better in plot]. Below, the separate segments are annotated to indicate pitch “targets” (coral vertical lines).

The recorded sound files were first processed with Praat (Boersma, 2001) to extract pitch and intensity values with a sample rate of 500 samples per second. Additional details can be found in Verhoef (2013). The resulting pitch and intensity tracks were then used for further processing in R. Whistle sounds often consisted of sequences of sounding and silent parts and the individual whistles were segmented into whistle segments, by finding silent breaks using the intensity track. These whistle segments were then further processed to find pitch targets, defined as the peaks and valleys in the contour, measured from the zero-crossings of the derivative of the pitch track. Figure 3 shows an example of one whistle segmented into sounding and silent parts followed by each sounding segment annotated with pitch targets.

### Measures

To find out if we see the emergence of musical features in these transmitted “proto-melodies,” we defined several measures to look for evidence of presence of the statistical universals in the pitch domain as described by Savage et al. (2015). Below we describe how we measured each of the seven Melodic Universals (MU).

#### MU1: Discrete Pitches

Over generations we look for a shift from a more continuous use of the signal space, to a more discretized use of tones or pitch level targets. To do this we take the locations of the targets found in the preprocessing steps (indicated in coral vertical lines in Figure 3) and list the semitone values at those locations for all whistles in a system of 12 whistles (one generation in each chain). We first look at the distributions of these tone targets to see if they become increasingly less uniform towards the end of the chains. This is achieved by running Chi-square (χ^2^) goodness-of-fit tests, comparing each system to a uniform distribution. Then, we test whether the distributions become more “peaky,” meaning that there are several clear, more discrete, targets. By modelling the continuous, unrounded data with Gaussian Mixture models (using the “Rmixmod” library in R by Langrognet et al., 2020) we find the best fitting model for each generation in each chain and extract the number of clusters found for that model. Each run on the same data does not always find the same model to be the best, so we run it 100 times on each chain/generation and take the average. If the best fitting models find on average more clusters, this indicates the distribution is more peaky and has more well-defined tone targets.

#### MU2: Few Elements

The discrete pitch targets of the whistle systems should also get organised in scales of few (≤ 7) elements per octave. To determine this we use the same data that was generated for MU1 to find out if the number of clusters that are found obeys this constraint.

#### MU3: Descending or Arched Contours

The melodies created by the participants should primarily show simple descending or arched contours. To test this we analyse the number of tone targets in each segment to see whether later systems produce less complex contours, with fewer up and down movements. We also look at the contour types in more detail by first categorising each segment based on the number of targets and the tone heights and differences of those targets into one of the following categories (Savage et al., 2020):

- “Horizontal”–no ascending or descending slides
- “Ascending”–ascending slides only
- “Descending”–descending slides only
- “Arched”–first ascending, then descending
- “U-shaped”–first descending, then ascending
- “Undulating”–multiple changes of direction

Then, we measure the proportion of total use for each type to see if a preference for descending and arched contours appears over time.

#### MU4: Small Frequency Intervals

**Figure 4:**
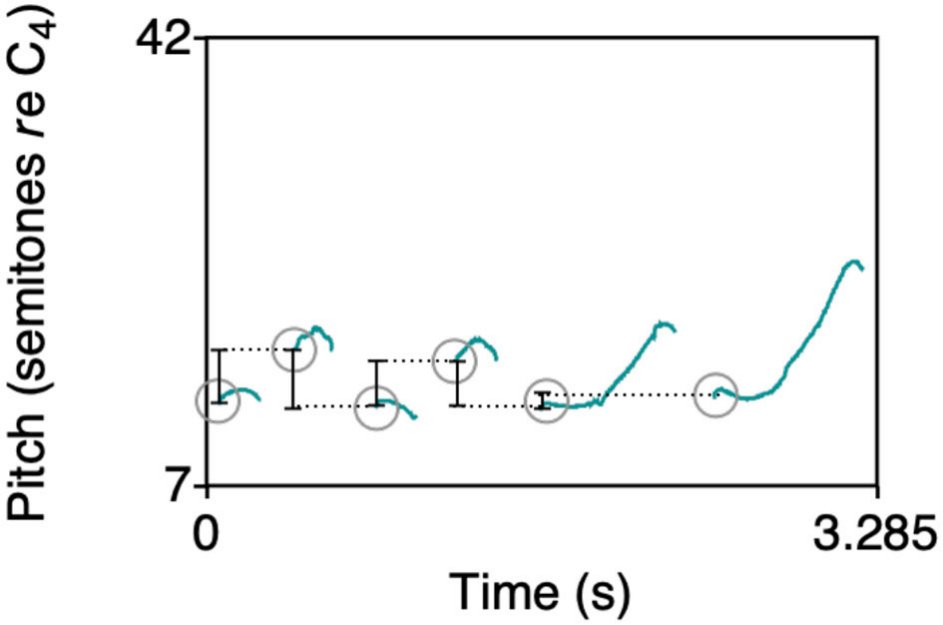
A whistle, plotted as pitch track on a semitone scale (green lines), where onsets are indicated in grey circles, and black lines show onset-to-onset intervals. For computing the interval compression ratio, the individual segments in each whistle are shuffled 100 times and the mean absolute differences between shuffled onsets are computed.

Melody contours in music typically span small frequency intervals (≤ 750 cents, i.e., around a musical fifth). To see if this is the case for the produced whistle melodies too, we compute the onset-to-onset intervals (illustrated in Figure 4) in semitones for consecutive segments in each whistle and check whether the average interval size between two consecutive onsets gets reduced in size towards later generations in a chain. In addition we look at interval compression ratios (Tierney et al., 2011; also used by Lumaca and Baggio, 2016) which provides another way to see if the melodies become more compressed. This measure compares the actual interval sizes to the intervals between segment onsets if the segments within a whistle are randomly shuffled (repeated 100 times). So we take the mean absolute differences between onsets of a whistle where the segments are randomly shuffled and divide it by the actual mean interval size in the whistle. This is the interval compression ratio.

#### MU5 and MU6: Motivic Patterns and Repetition

The whistled systems that emerge over generations are expected to increasingly exhibit motivic patterns and repetition. This set of musical universals is structurally similar to the combinatorial structure and phonological patterning that was the focus of the original study for which the whistle data was collected. In the work by Verhoef et al. (2014) two relevant measures were reported and will be reused here. The first involves determining a set of basic building blocks in each whistle system and showing that the sets in later generations were composed of a smaller set of basic building blocks that were increasingly repeated and combined. This was measured with Shannon entropy (Shannon, 1948). The segments that were found in the preprocessing steps were clustered for each set of 12 whistles, using average linkage agglomerative hierarchical clustering (Duda et al., 2001) to group together segments that were similar enough to be considered the same category or building block. Segment similarity was measured by comparing movement patterns through the pitch space using Derivative Dynamic Time Warping (Keogh and Pazzani, 2001). Clustering continued until there was no pair of segments left with a distance smaller than 0.08. The following equation from Shannon (1948) was used to compute entropy, where *p*_*i*_ is the probability of occurrence of building block *i*, measured as their relative frequencies:

$$\begin{array}{l} H=-\sum p_{i}\log p_{i} \end{array}$$

As a second measure to test for the presence of motivic patterns and repetition, we look at the associative chunk strength (Knowlton and Squire, 1994), which calculates the sequential and adjacency structure of building blocks and originates from the field of artificial grammar learning. This measure is computed by using the building blocks that were found as described above for measuring entropy. All bigrams and trigrams of building blocks that occurred in the whistles were identified and their frequencies in the whistle sets were counted. The associative chunk strength of a whistle set is the average of the bigram and trigram frequencies.

#### MU7: Short Phrases

Phrases in the whistle melodies should be short (≤ 9 s). This last one is harder to show in our dataset since the whistle signals fed to the first-generation participants are all shorter than 9 s. The longest whistle lasts 8.4 s and the mean whistle duration is 2.5 s, while the mean whistle segment duration is 0.5 s. However, musical phrases consist of motifs and other more basic elements, and we can test (1) whether we see changes over generations in the number of segments per whistle, and (2) whether the whistles become shorter overall, or instead, (3) whether they become longer approaching a theoretical asymptote of 9 s.

In the following paragraphs we present the results, where all of the measures described above were analysed using linear mixed-effects models. All analyses were conducted in R (R Core Team, 2019), using the packages lmerTest (Kuznetsova et al., 2017), lme4 (Bates et al., 2015), and tidyverse (Wickham et al., 2019). *P*-values for fixed effects were calculated with *t*-tests using Satterthwaite's method (Kuznetsova et al., 2017). Random intercepts were included for Chain and no random slopes were added.

## Results

### MU1: Discrete Pitches

**Figure 5:**
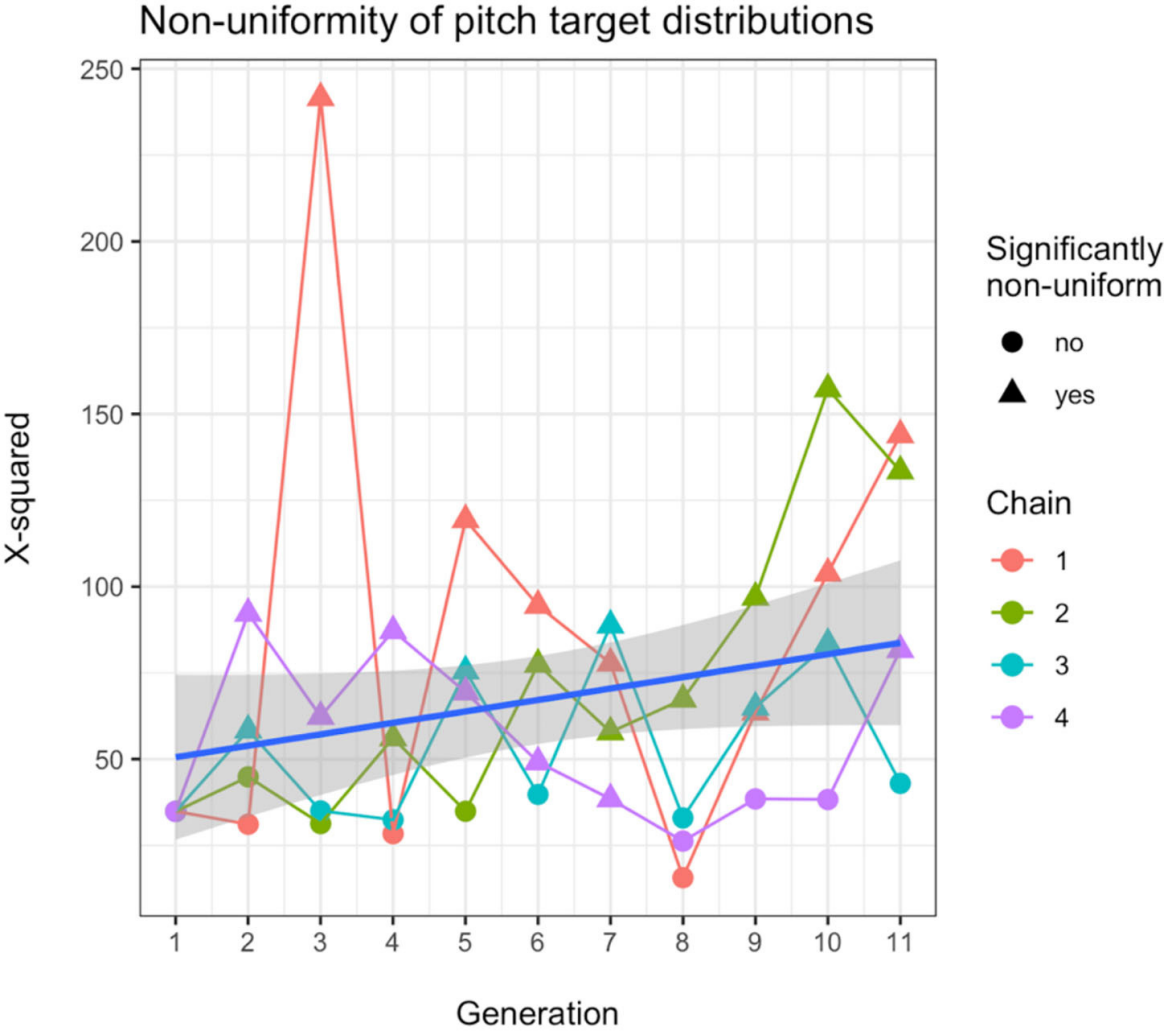
Chi-square (χ^2^) statistic measured for each generation in each chain to test for non-uniformity of tone target distributions. The plotted shape of the datapoint indicates whether the tone target distribution is significantly non-uniform (*p* < 0.001). The blue line shows a linear model fit over all data and the grey area is the 95% confidence level interval.

**Figure 6:**
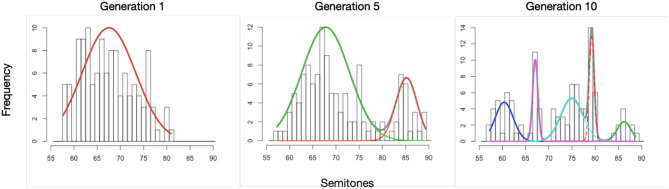
Example results of the Gaussian Mixture models, used to measure discretization of the continuous pitch range into clusters of tone targets. These figures show the best fitting model for generation 1, 5, and 10 for one chain. More and clearer clusters are found in the less uniform later distribution.

**Figure 7:**
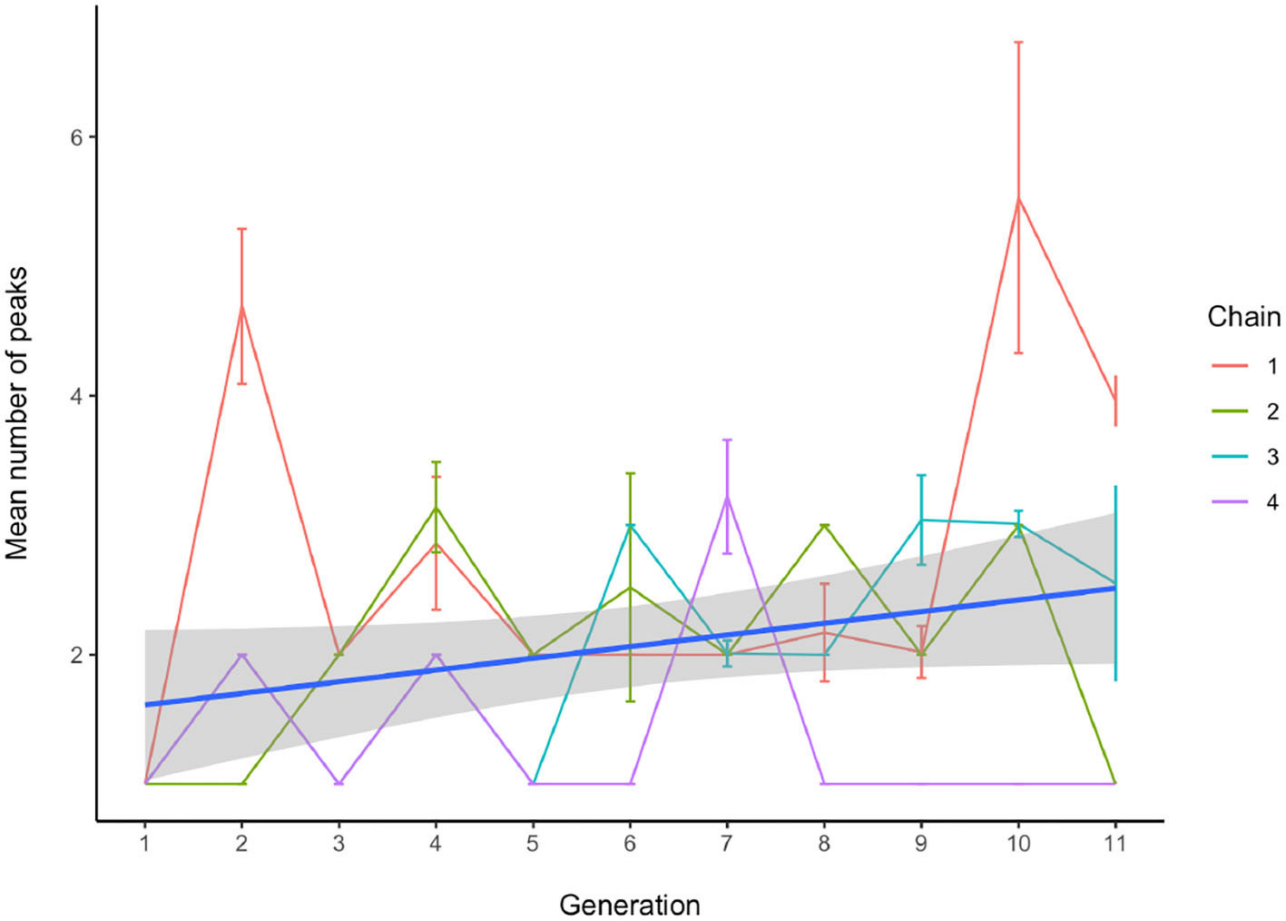
The number of whistle tone clusters found by the Gaussian Mixture models. Earlier productions tend to exhibit more continuous use of the pitch space, while in later generations, more distinct clusters are found. Even though results vary per chain, overall a significant increase in the number of discrete tone categories is found.

First, we test whether the uniformity of the distribution of tone targets changes over generations. We use the Chi-square (χ^2^) statistic, computed to determine for each whistle system whether its tone targets are distributed in a way that is significantly different from uniform. Higher values are associated with distributions closer to being significantly non-uniform (assuming the same number of degrees of freedom, which is approximately the case here across systems with a standard deviation of 2.4). With a linear mixed-effects model we find that the measured χ^2^ statistics are affected by generation, with a small effect which is marginally significant (*b* = 3.314 ± 1.962 SEM, *P* = 0.099), where this value increases on average by 3.3 for each generation. Figure 5 shows the development of the χ^2^ values for all chains, where the shape of the point indicates whether the *P*-value of the test was below 0.001, as well as an overall linear model fit. The results of the model and the figure indicate that the distributions of tones become less uniform over time. To see if they also become more peaky, with several clear tone targets, we modelled the number of categories that were found by the Gaussian Mixture models as a function of generation and found that the average number of peaks increased significantly over time (*b* = 0.090 ± 0.043 SEM, *P* = 0.04). Figure 6 shows examples of early, middle and late generation distributions with fitted gaussians and Figure 7 plots the number of categories found for each chain over generations.

### MU2: Few Elements

Typically, discrete tones in musical systems are organised in such a way that there are few (≤ 7) elements per octave. If we look at the number of peaks which was used to identify discrete tone categories in the whistle systems, we see that the maximum number found was 5 (generation 10, chain 1), which is smaller than 7. On average the number of peaks was 2 (sd = 1.01), including all generations, and for the initial whistle set it was 1. Determining whether this gradually becomes more music-like in our data is harder in this case since the actual discrete pitches had not emerged yet in the initial whistle sets, but we do see that, while the pitch target distributions become less uniform (as shown in MU1), a number of categories emerge in each set that are never above the numbers typically found in real musical systems. While it is hard to know for sure whether this growth would level off if the transmission chain would continue, we do see that generally in these kinds of transmission experiments the most substantial changes happen within the first 10 generations (e.g., Claidière et al., 2014), as is also the case for several other measures discussed in this paper.

### MU3: Descending or Arched Contours

**Figure 8:**
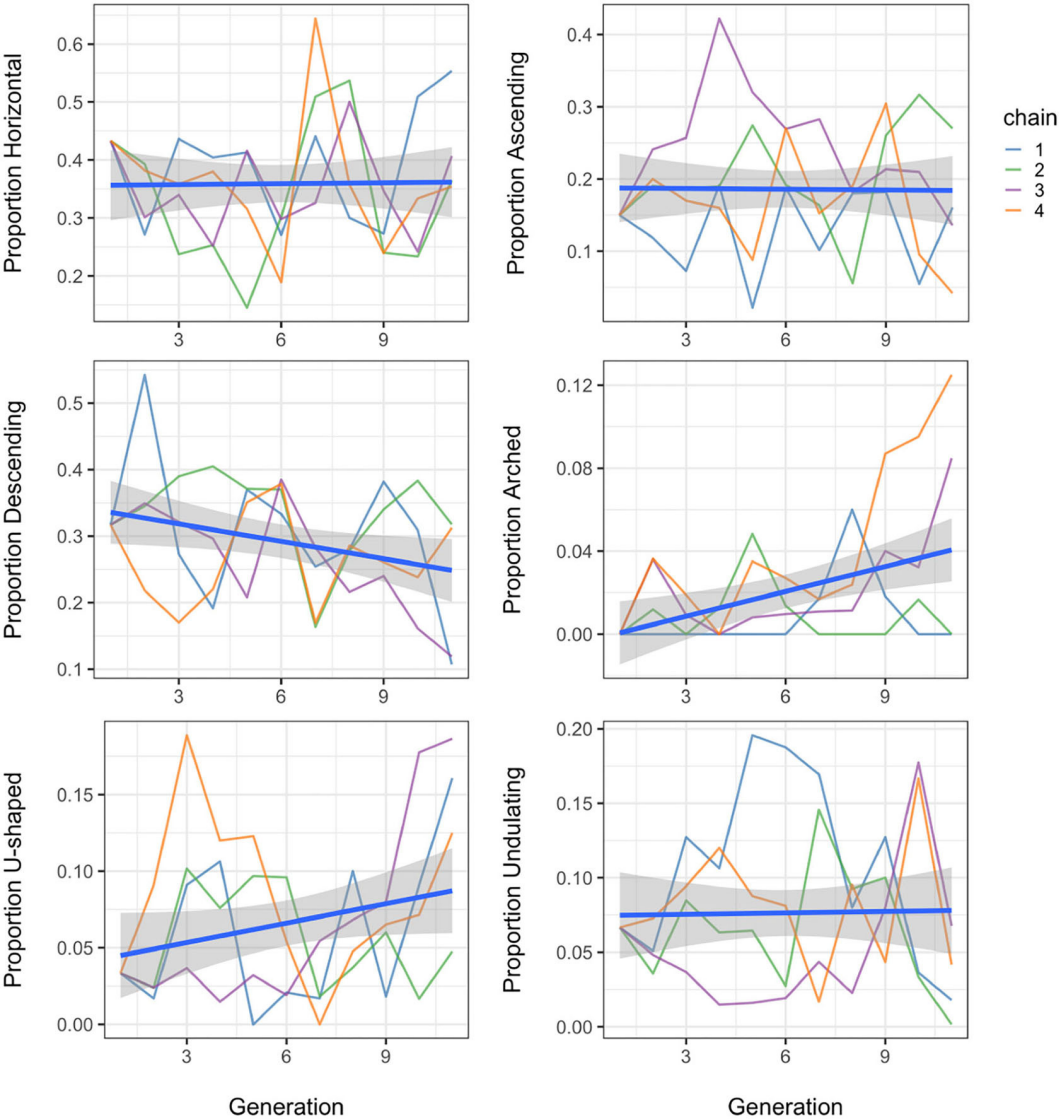
Distribution of segment types over generations, showing the proportion of each type in the four chains, over generations. Each whistle segment has been categorised as horizontal if there are no ascending or descending slides, ascending if it has ascending slides only, descending if it has descending slides only, arched for first ascending, then descending, u-shaped for first descending, then ascending and undulating in case of multiple changes of direction.

We tested whether the melodies created by the participants primarily show contour types that are simple and in particular of the descending or arched types. To test this, we first assessed the overall complexity of the contours by modelling the number of tone targets per segment as a function of generation. Results show that the later generations produce contours that contain fewer up and down movements (*b* = −0.020 ± 0.008 SEM, *P* = 0.03), with on average 0.02 fewer tone targets by each increasing generation. Contours became gradually less complex towards later generations in a chain. We also compared the prevalence of descending and arched contours to that of the other non-monotonic types and modelled the proportion used for each type in the whistle systems as a function of generation. Here we found no significant effect of generation for horizontal, ascending, u-shaped and undulating contours, but there was a significant effect of generation on the use of arched contours (*b* = 0.004 ± 0.001 SEM, *P* = 0.001), as well as for the use of descending contours (*b* = −0.009 ± 0.004 SEM, *P* = 0.03). However, the statistical effect is in the expected direction only for the arched contours; the descending contours have a slight but significant decrease in use instead of an increase. It is worth mentioning though that the initial whistle set had a relatively large proportion of descending contours already (0.32), so these tended to be preferred in systems in general, while the initial set contained no arched contours at all, which were therefore spontaneously introduced. Overall, horizontal and descending contours are the most prevalent, together consistently making up more than half (and up to 70%) of the segments across generations. Figure 8 shows the development of the proportion each contour type took in the whistle sets over generations.

### MU4: Small Frequency Intervals

**Figure 9:**
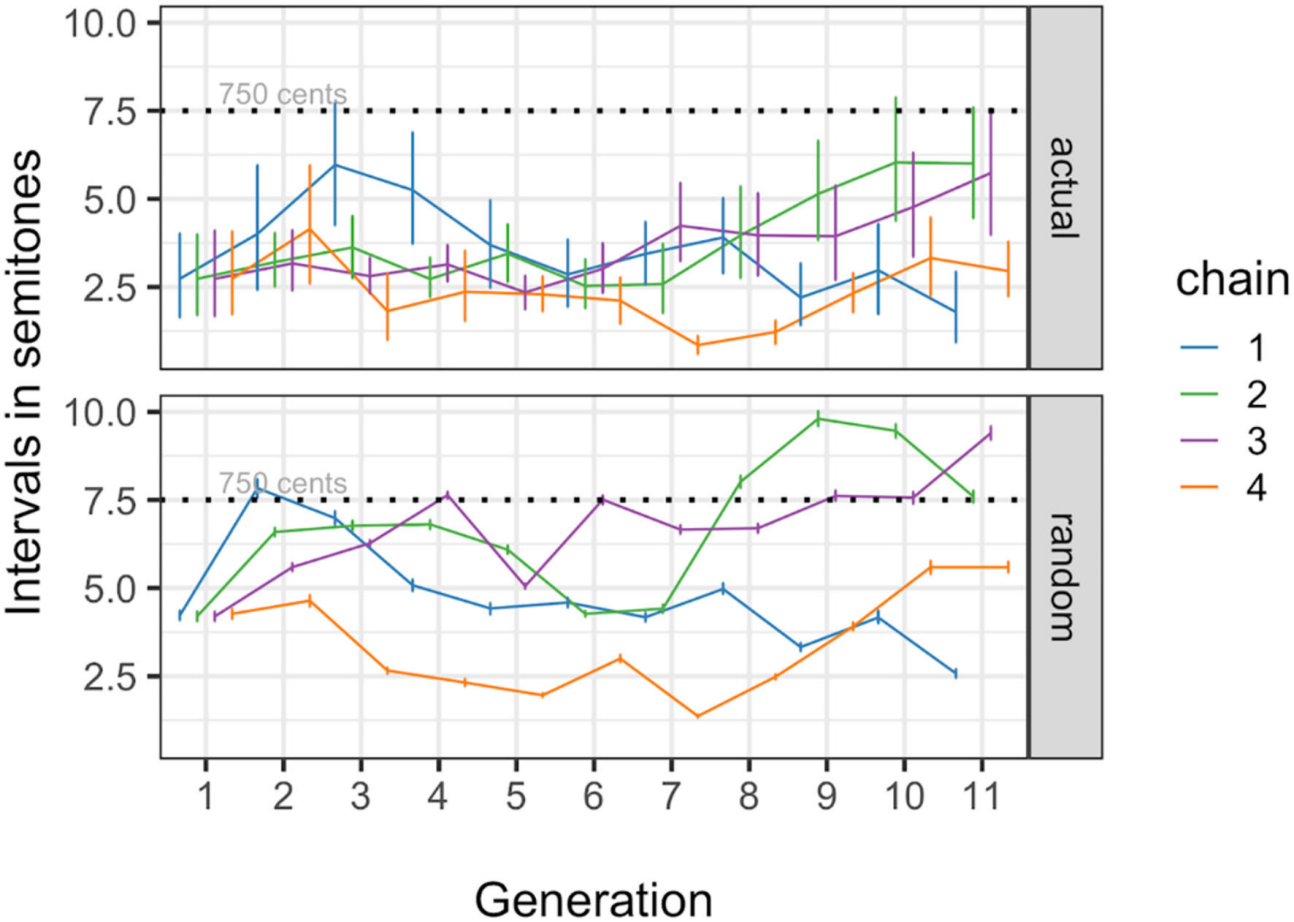
Mean absolute onset-to-onset differences across chains and generations for the actual data (top panel), as well as means for the intervals measured in 100 randomly shuffled whistles (bottom panel). The dashed line indicates the 750 cent level.

To test if the evolved whistle melody contours span small frequency intervals (≤ 750 cents), we modelled the onset-to-onset intervals in semitones for consecutive segments in each whistle as a function of generation. Results show that there is a main effect of generation on the intervals (*b* = 0.103 ± 0.028 SEM, *P* < 0.001), but it is in the opposite direction of what we expected, where intervals are getting on average 0.1 semitones larger by each increasing generation. Overall, however, interval sizes are clearly lower than 750 cents, already from the start and throughout the experiment. In addition we compared the actual interval sizes to intervals computed for whistles with randomly shuffled onsets and Figure 9 shows the mean absolute onset-to-onset differences across chains and generations as well as means for the intervals measured in the 100 randomly shuffled versions of the whistles. The dashed line indicates the 750 cent level. As a way to see if the melodies become more compressed over generations, the interval compression ratio (random intervals/actual intervals) was modelled as a function of generation and dataset (actual vs. random), and we found a very strong effect of dataset on the intervals, where the onset-to-onset interval in semitones is overall smaller in the actual whistle data as compared to the data in which the segment onsets are shuffled in each whistle (*b* = 2.306 ± 0.236 SEM, *P* < 0.001). We also find a main effect of generation, but as expected based on the model that included only the actual data, the effect is in the opposite direction of what we expected, where intervals are getting on average 0.11 semitones larger by each increasing generation (*b* = 0.110 ± 0.036 SEM, *P* = 0.002). There is no interaction between generation and dataset (*b* = 0.011 ± 0.036 SEM, *P* = 0.75). The results are shown in Figure 9 as well: a small increase in interval size can be seen, while the vast majority of the actual data has intervals that remain below 750 cents, and in random data the 750 cents threshold is more often surpassed.

### MU5 and MU6: Motivic Patterns and Repetition

**Figure 10:**
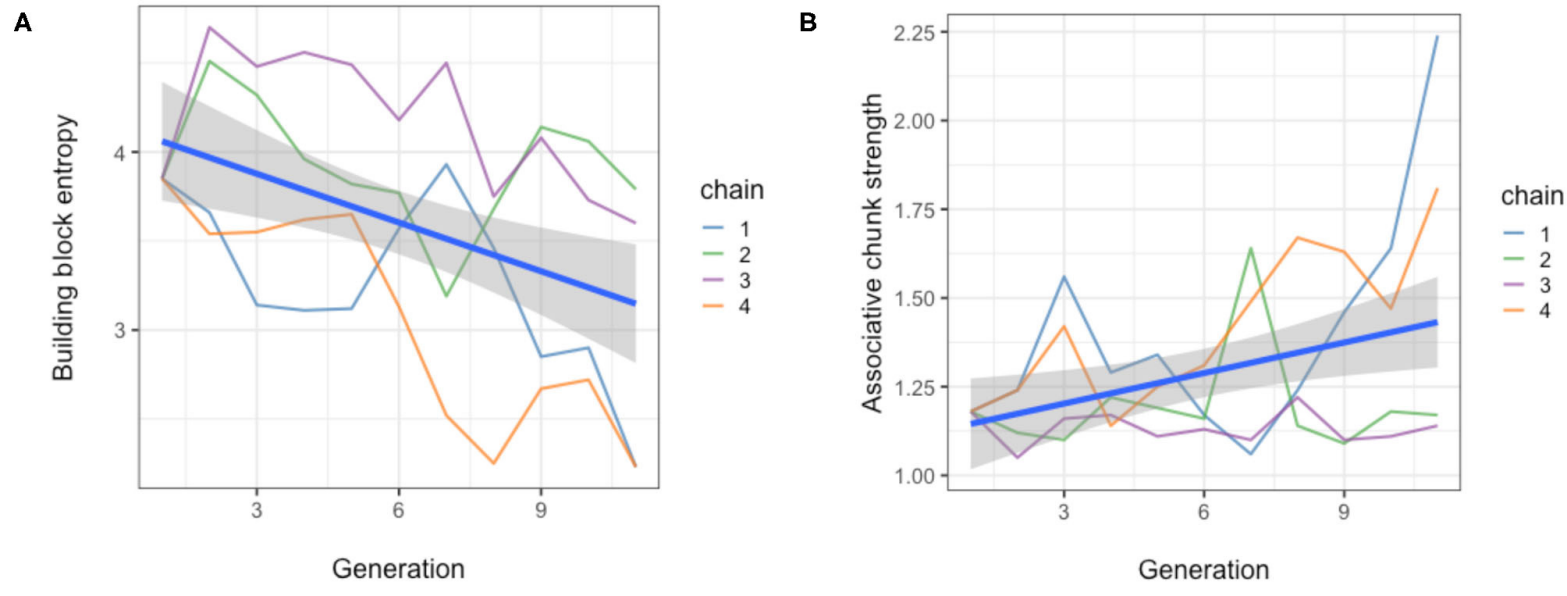
Two measures demonstrating the emergence of motivic patterns and repetition. **(A)** Entropy of the whistle sets over generations for all four chains, demonstrating that structure and reuse of elements increases. **(B)** Associative chunk strength of the whistle sets over generations for all four chains, showing an increase in reoccurrences of bigram and trigram sequences of basic whistle elements, and thereby demonstrating the emergence of sequential structure. The blue lines show linear model fits and the grey areas are the 95% confidence intervals.

The whistled systems were analysed to test whether they increasingly exhibit motivic patterns and repetition. The first measure computed Shannon entropy (Shannon, 1948) over the whistle building blocks (clustered segments), and these entropy values were modelled as a function of generation to see if entropy decreased (meaning that compressibility and structure increased). We found a significant effect of generation on building block entropy (*b* = −0.091 ± 0.018 SEM, *P* < 0.001), with entropy decreasing by nearly 0.1 per increasing generation. This implies a gradual increase of compressibility and reuse of elements. These results are shown in Figure 10A.

We also measured the associative chunk strength, as a measure of sequential structure and modelled this as a function of generation as well. We found that generation significantly affects associative chunk strength (*b* = 0.028 ± 0.009 SEM, *P* = 0.007), with an increase in this value by nearly 0.3 per increasing generation. This suggests there is a trend towards increasing sequential structure and patterning of elements. These results are shown in Figure 10B.

**Figure 11:**
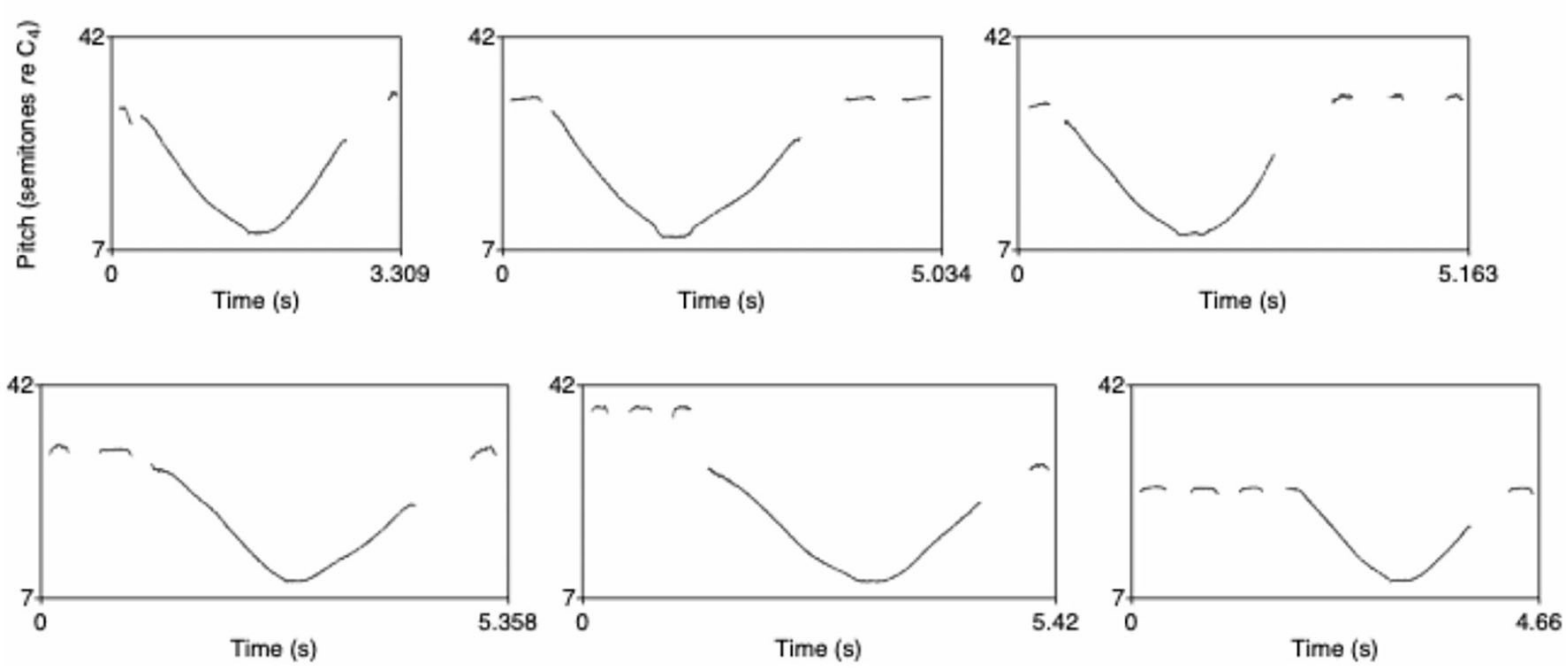
Fragment of the whistles plotted as pitch tracks on a semitone scale in the last set of a chain. Basic elements can be identified that are systematically recombined. Reprinted with permission from Verhoef (2012).

Figure 11 shows an example fragment of a last-generation whistle set to illustrate these findings. In this set we can identify clear repetition and patterning. There is a small set of basic building blocks (arched contours and short level notes) and these are reused and combined in different but systematic ways to create the whistles in the set (from Verhoef, 2012).

### MU7: Short Phrases

As mentioned in the methods section, the whistle signals are all shorter than 9 s already, but we tested for changes over generations to see if the number of segments per whistle shortens and the total whistle durations become shorter overall. Therefore we first modelled the number of segments per whistle as a function of generation and found that indeed whistle phrases increasingly use fewer segments over generations (*b* = −0.123 ± 0.056 SEM, *P* = 0.03). As for the total whistle durations, we find no change towards shorter whistles, in fact there is a slight, marginally significant effect in the opposite direction towards longer (though still shorter than 9 s) whistles (*b* = 0.045 ± 0.024 SEM, *P* = 0.07), with whistles becoming 0.045 s longer on average for each generation.

## Discussion

This research was conducted with the goal of testing whether statistical melodic universals can emerge via human (cognitive, motoric, etc.) biases that are amplified and modulated by cultural transmission. We used data from an experiment that simulated cultural transmission of whistled systems, where sets of whistle sounds were repeatedly learned and reproduced by different individuals in transmission chains (Verhoef, 2012). A large body of work on such iterated learning experiments has shown that transmitted systems tend to gradually change and adapt to the cognitive biases of the learners (Kirby et al., 2008, 2014). Here, we used the emerged whistle systems to quantify a subset of the universal features in music described by Savage et al. (2015) and tracked the emergence of these features. In particular we studied seven melodic universals: a transition from continuous to discrete pitches (MU1), which are organised in scales of few (≤ 7) elements (MU2); whether melodic elements constructed from these pitches show primarily descending or arched contours (MU3), and melody contours span small frequency intervals (MU4) as well as the presence of motivic patterns (MU5), repetition (MU6), and short phrases (MU7).

For MU1 we found, as expected, that the distributions of pitches used in the whistles produced by participants become less uniform over time, where the number of discrete pitches increases, resulting in more peaky distributions; notice however that in our case, and unlike common musical systems, there were few rather than several peaks within octaves. This could therefore be the start of a discretization process that would eventually result in a fixed set of tone categories with fixed intervals. We need to point out though that finding the type of fine-grained discretization of a fully developed musical system was not the goal here since our participants were not musicians and did not master the slide whistle instrument professionally. Therefore, it would not have been possible for them to hit target notes as precisely as trained musicians. What we were looking for and found was initial evidence of a discrete set of preferred pitch areas that was used repeatedly across a whistle set. We also found that the number of discrete pitch categories, while increasing, remained below 7 elements (MU2) consistently across chains and over generations. As for the contour types (MU3) we see a general trend towards less complex contours emerging over generations in the chains. Results are mixed for descending and arched contours. We do see a significant relative increase in arched contours, but the use of descending contours shows a slight decrease over time. However, as mentioned before, the descending contours were actually quite heavily overrepresented in the initial set so there seems to be a clear preference for those contours throughout the experiment and they kept being used relatively often until the last generation. There were no arched contours in the initial set, so the fact that arched contours were introduced by participants and increased over time with no prior example is suggestive (but note that arched contours remain rare throughout). This combined with the fact that all other types of contours showed no change over time, gives us at least some evidence that this universal could also emerge. Looking at the interval sizes of melodic contours (MU4), we find that the onset-to-onset intervals, measuring the distance between the starting tones of consecutive segments, increase over time. Although this seems to contradict the general expected direction of intervals becoming smaller, we do see that they actually get closer to the interval size that is often found in musical systems (≤ 750 cents). We also find that across chains and generations, the onset-to-onset intervals are smaller in the actual whistle data as compared to the data in which the segment onsets are shuffled in each whistle, which is also the case for real music (Tierney et al., 2011). In addition, we found an increase in motivic patterns (MU5) and repetition (MU6), measured as a decrease in the entropy of basic building block use and increase of frequencies of recurring bigrams and trigrams of elements. Finally, whistles contained short phrases (MU7). Throughout the experiment, even though the duration did not decrease over time, the whistle sounds were shorter than or almost equal to the duration typically found in musical phrases (<9 s).

Altogether we can conclude that most of the seven features we tested could be found, wholly or partially, in the transmitted whistle sets. For some of the measures where a change over generations was not found, it often was the case that the structure or feature was already present in the initial set and therefore relatively stable throughout generations. This initial set was designed to study the emergence of combinatorial structure in language and was deliberately created in such a way that it did not have any obvious combinatorial structure, but for most of the musical features we studied here, this was not taken into account. Perhaps stronger effects over generations could be found with initial sets where interval sizes, phrase lengths and distributions of contour types are deliberately made less music-like. In fact, we suggest that future work could provide first-generation participants with initial sets that either lack (e.g., no arched contours) or over-represent (e.g., more than seven elements) musical universals; if cultural transmission and biases play such strong role, one would witness an increase in the former case and a decrease in the latter case. More generally, we recognise that a number of factors might modulate our results if our experiment were repeated; these include the effect of a different initial set of whistles, a greater number of chains, or more generations per chain. Results from this limited data set already allow for some interesting conclusions, but more work is needed to test for robustness of results as a function of the experimental parameters space.

A potential shortcoming of our results lies in the previous musical exposure of participants. Because of the testing locations, all participants already had some exposure to the Western musical tradition before the experiment. Due to this, participants' reproductions may have been affected by culturally-acquired priors on melodies. Nonetheless, we still believe this methodology provides a way of testing melodic universals, as long as experiments are repeated in several locations and cultures. Emergent properties in the melody chains may be culturally specific but every possible participant in the world belongs to a culture; what we believe is remarkable is that sampling even only part of the Western musical culture, like we did, still produces convergence to the statistical universals appearing above chance in most cultures.

Crucially, participants in this experiment were told they were learning a whistled language, not a musical system. It is therefore quite remarkable that we still find evidence for musical universals emerging in these systems. However, the slide whistle is a musical instrument with intrinsic musical qualities and participants may have been biassed towards creating music-like signals. This may explain why some musical universals we measured were already present in the first generation of the transmission chains. As Ma et al. (2019) have shown, transmission chains of vocalisations diverge into more music-like forms when they refer to emotions, while they become more speech-like when they refer to a concrete object. The slide whistle signals that were transmitted in our experiment were not connected to any specified referent and they needed to be distinct “words,” which created a pressure for expressivity (Kirby et al., 2015) and may have pushed the system more towards language-like signals. In any case, not all musical universals were present from the start, but many gradually appeared after repeated transmission events, showing that most of these universals were not automatically connected to the use of a musical instrument. The fact that musical universals emerge even in the case of a language learning task illustrates both the robustness of the emergence of these types of structure and also the fact that some structural similarities exist between language and music. The entropy-based measure for motivic patterns (MU5), for instance, is directly equivalent to the measure of phonemic patterning used by Verhoef et al. (2014), illustrating such parallels in structure.

To conclude, we presented evidence that, besides the earlier studied rhythmic universals (Ravignani et al., 2016), also some melodic universals can evolve by adaptation to human biases over generations of cultural transmission.

## Data Availability Statement

The raw data supporting the conclusions of this article will be made available by the authors, without undue reservation.

## Ethics Statement

The studies involving human participants were reviewed and approved by UCSD's Human Research Protections Program as well as by the Commissie Ethiek of the Faculteit der Geesteswetenschappen at the University of Amsterdam. The participants provided their written informed consent to participate in this study.

## Author Contributions

TV designed the experiment, collected the data, and conducted the analyses. AR and TV designed the analyses and wrote the paper. All authors contributed to the article and approved the submitted version.

## Conflict of Interest

The authors declare that the research was conducted in the absence of any commercial or financial relationships that could be construed as a potential conflict of interest.

## Publisher's Note

All claims expressed in this article are solely those of the authors and do not necessarily represent those of their affiliated organizations, or those of the publisher, the editors and the reviewers. Any product that may be evaluated in this article, or claim that may be made by its manufacturer, is not guaranteed or endorsed by the publisher.
